# Radiation‐Triggered Chemokine‐Secreting Probiotics for Boron Neutron Capture Therapy Based Radioimmunotherapy

**DOI:** 10.1002/advs.77542

**Published:** 2026-09-01

**Authors:** Yang Liu, Yuzhong Qian, Shaobo Huang, Bingbing Li, Yue Yu, Xiang Cheng, Chang Chen, Jiheng Wang, Maosong Yang, Lizheng Liang, Xiancai Meng, Zhigang Liu

**Affiliations:** ^1^ Institute of Energy Hefei Comprehensive National Science Center（Anhui Energy Laboratory） Hefei Anhui China; ^2^ Anhui University of Science and Technology Huainan China; ^3^ Cancer Center Dongguan Key Laboratory of Precision Diagnosis and Treatment for Tumors Guangdong Engineering Research Center of Boron Neutron Therapy and Application in Malignant Tumors The Tenth Affiliated Hospital Southern Medical University (Dongguan People's Hospital) Dongguan China; ^4^ Hefei Cancer Hospital Chinese Academy of Sciences Hefei China; ^5^ State Key Laboratory of Pharmaceutical Biotechnology Division of Hepatobiliary and Transplantation Surgery Department of General Surgery Nanjing Drum Tower Hospital Affiliated Hospital of Medical School Nanjing University Nanjing China

**Keywords:** boron neutron capture therapy, probiotics, radioimmunotherapy

## Abstract

An ideal radioimmunotherapy eradicates primary tumors and drives immune clearance of residual or metastatic tumor cells, but its clinical translation is severely limited by systemic immune toxicity and the immunosuppressive tumor microenvironment (TME). Here, we constructed engineered Escherichia coli Nissle 1917 with a tannic acid‐mediated boronophenylalanine coating and a radiation‐responsive genetic circuit, enabling TME‐responsive boron delivery for boron neutron capture therapy (BNCT). BNCT‐mediated selective tumor killing spared intratumoral probiotics, whose integrated genetic circuit was triggered by the low‐level incidental radiation generated during the BNCT process, including γ‐rays, to drive CXCL10 production, forming a feedback loop to remodel the TME. This strategy achieved robust tumor eradication, inhibited distal tumor growth and post‐surgical metastasis in murine models, pioneering a BNCT‐based radioimmunotherapy modality.

## Graphical abstract

Engineered Escherichia coli Nissle 1917 with a tannic acid‐mediated BPA coating enables tumor‐targeted boron delivery for BNCT. The low‐level incidental radiation generated during BNCT activate a RecA‐driven circuit to induce CXCL10, while radiation‐enhanced bacterial colonization triggers in situ vaccination and systemic antitumor immunity.

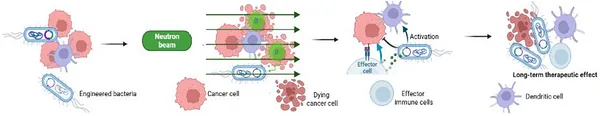

## 1 Introduction

Cancer immunotherapy has revolutionized clinical oncology, yet durable responses in most solid tumors remain severely limited by an immunosuppressive tumor microenvironment (TME) and insufficient priming of tumor‐specific adaptive immunity [1, 2, 3, 4]. Radiotherapy is widely applied to potentiate immunotherapeutic efficacy [5, 6, 7], but it is trapped in a fundamental and unresolved therapeutic paradox. High radiation doses are required to drive effective tumor eradication, a process that provides a critical time window for adaptive immune priming [7, 8]. These same doses consistently compromise functional immune populations [9, 10], worsen the immunosuppressive features of the TME, and drive tumor immune escape and metastatic spread [11, 12].

To mitigate this long‐standing paradox, extensive preclinical investigations have combined conventional photon radiotherapy (X‑ray or γ‑ray) with bacteria‑mediated tumor therapy. Anaerobic and facultative anaerobic strains, including Clostridium, Salmonella typhimurium, and Bifidobacterium, exhibit inherent tropism for hypoxic tumor niches. Spores of Clostridium species have been well characterized as tumor‐specific delivery vehicles with selective germination and oncolytic activity in hypoxic tumor regions [13]. The tumor‐homing specificity of attenuated S. typhimurium has been further validated by in vivo molecular imaging studies [14] and engineered Bifidobacterium longum has been exploited as a targeted vector for therapeutic gene delivery [15]. When combined with radiotherapy, these bacterial vectors have been reported to enhance local tumor control via direct bacterial oncolysis and complementary immune stimulation [16]. Nevertheless, this combined modality still confronts three major unresolved challenges that substantially restrict its clinical translatability. First, bacterial radiosensitivity: conventional high‑energy photon radiation delivers non‑selective, deep‑penetrating doses that exert direct DNA damage to therapeutic bacteria, reducing their viability and proliferative capacity. For facultative anaerobic strains such as attenuated Salmonella typhimurium, ionizing radiation at clinically relevant doses has been documented to impair intratumoral bacterial survival, which has motivated efforts to engineer radiation‑tolerant bacterial strains for combined therapy [17]. For obligate anaerobic species including Bifidobacterium and Clostridium, their germination and colonization are strictly dependent on the hypoxic tumor niche [18]. Radiotherapy‑induced transient tumor reoxygenation disrupts this essential microenvironment, markedly diminishing their tumor‑targeting efficiency and sustained oncolytic effects.

Second, insufficient immune activation: despite the intrinsic adjuvant activity of bacteria, high‑dose conventional radiotherapy aggravates the immunosuppressive TME by recruiting myeloid‑derived suppressor cells (MDSCs) and regulatory T cells (Tregs), and upregulating inhibitory molecules such as PD‑L1 and TGF‑β. These effects largely counteract bacteria‑mediated immune priming. Clinical data consistently show that intratumoral administration of anaerobic bacteria (e.g., Clostridium novyi‑NT) only enhances local tumor‑infiltrating lymphocytes but fails to induce robust systemic anti‑tumor immunity or abscopal effects [19]. Preclinical studies further confirm that even when engineered bacteria are applied to target immunosuppressive pathways, without specific intervention against radiotherapy‑induced inhibitory signals such as immune checkpoint upregulation, the functional activity of tumor‑specific T cells remains severely restricted, preventing the establishment of durable immune memory [20].

Third, dose‑limiting toxicity: escalating radiation doses to achieve synergistic tumor ablation inevitably increases injury to adjacent normal tissues. In preclinical mouse studies, single‐dose abdominal x‐ray irradiation at 13.5 Gy consistently induces severe radiation enteritis (equivalent to grade 3‐injury), characterized by intestinal mucosal barrier disruption, crypt necrosis, and profound body weight loss. This is a well‐recognized severe normal tissue injury model in preclinical radiobiology, and corresponds to the dose‐limiting toxicity endpoint in combination regimens [21]. Meanwhile, systemic administration of therapeutic bacteria carries inherent risks of off‑target inflammation and bacteremia. The phase I clinical trial of attenuated S. typhimurium VNP20009 confirmed dose‑limiting toxicities including persistent fever, thrombocytopenia and systemic cytokine release at higher doses [22]. Notably, radiotherapy‑induced tissue barrier damage can further exacerbate bacterial translocation and systemic inflammatory risks, creating a superimposed toxicity that narrows the therapeutic window of the combination regimen.

Boron neutron capture therapy (BNCT) triggers nuclear fission of non‑radioactive ^10^B atoms via thermal neutrons, generating high‑linear energy transfer (high‑LET) α particles and ^7^Li nuclei with an ultra‑short path length less than 10 µM [23, 24, 25]. This mechanism enables precise tumor ablation while sparing intratumoral immune compartments, and elicits robust immunogenic cell death to drive systemic anti‑tumor immunity [26, 27]. Despite this potential, clinical translation of BNCT is critically limited by heterogeneous intratumoral delivery of boron agents [25, 28]. The only clinically approved boron agent ^10^B‑p‑boronophenylalanine (BPA) shows markedly poor accumulation in the hypoxic TME [29, 30, 31]. Engineered live bacteria [16, 32] represent an optimal, clinically validated platform to address this unmet need, with intrinsic tumor‑homing capacity to hypoxic lesions, potent immune adjuvant activity validated by decades of clinical BCG use [33, 34], and full synthetic programmability as tumor‑specific live drug delivery vehicles [35, 36, 37].

Here, we developed a BNCT platform using engineered probiotics to address limitations to the clinical application of conventional radioimmunotherapy and existing boron delivery regimens. We used the tumor‑homing, immunoadjuvant, and genetically programmable features of engineered probiotics to improve the uniformity of intratumoral boron agent distribution, and paired BNCT‑mediated tumor ablation with immunomodulation regulated by a radiation‑responsive synthetic circuit (Figure 1). This approach is designed to establish a self‑sustaining anti‑tumor immune cycle that addresses the three bottlenecks of conventional radiotherapy–bacteria combinations. It further amplifies circumvents bacterial radiosensitivity via the localized, cell‑level radiation effect of BNCT, which preserves intratumoral bacterial viability and colonization. It amplifies immune activation through the synergism of high‑LET‑induced immunogenic cell death and bacterial adjuvant effects, without exacerbating TME immunosuppression. Finally, it reduces dose‑limiting toxicity by restricting radiation damage to boron‑loaded tumor cells and limiting bacterial colonization to hypoxic tumor lesions. Collectively, this strategy provides a clinically translatable approach for combined radiotherapy and immunotherapy in refractory solid tumors.

**FIGURE 1:**
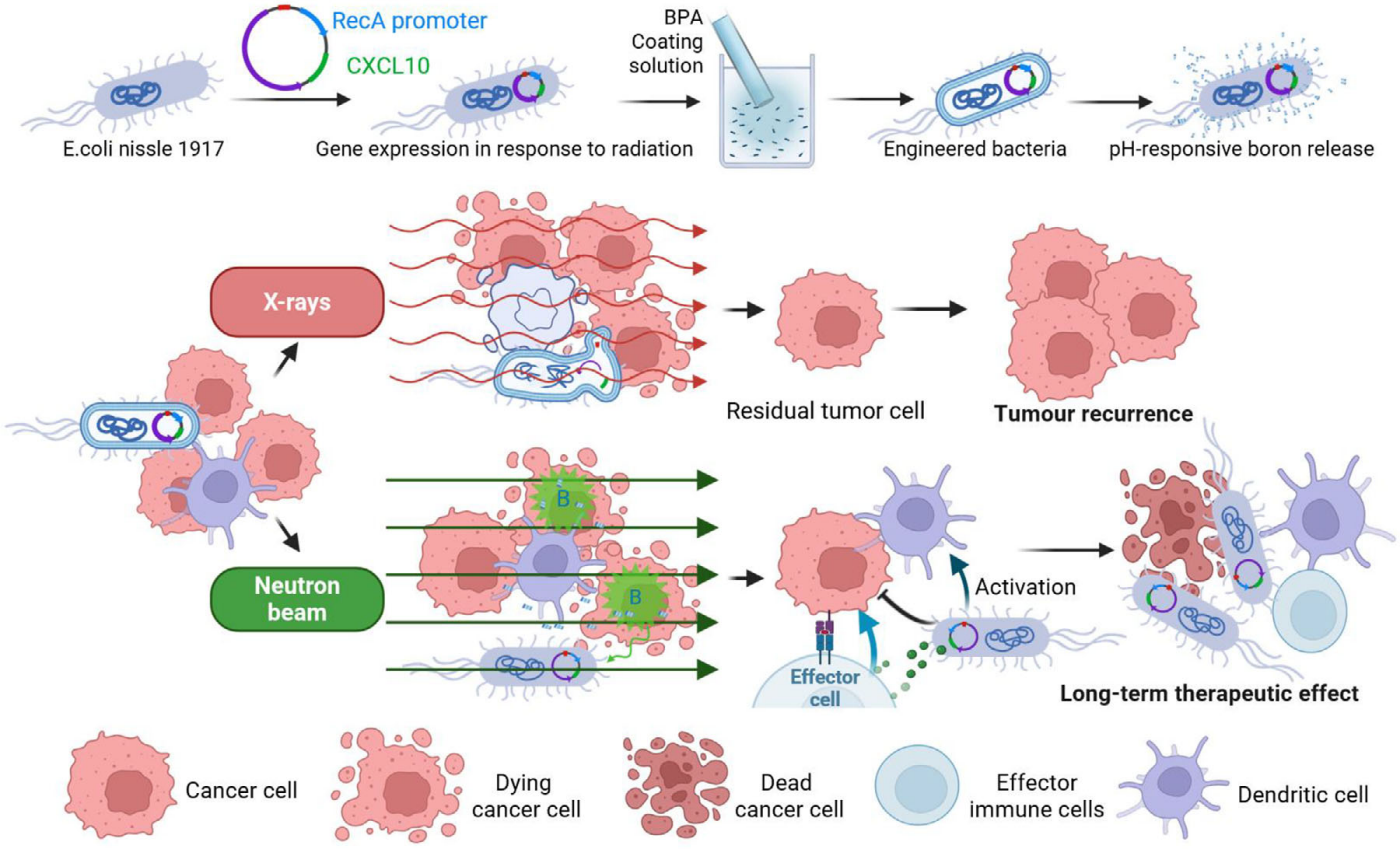
Engineered probiotic‐based boron delivery and radioimmunotherapy. Engineered *Escherichia coli* Nissle 1917 with a tannic acid‐mediated boronophenylalanine coating enables tumor microenvironment‐responsive boron release. During BNCT, low‐dose incidental radiation (including γ‐rays) activate a RecA promoter‐driven circuit to induce CXCL10 expression, while radiation‐induced tumor damage enhances intratumoral bacterial colonization. These mutually reinforcing effects amplify therapeutic protein production, driving an in situ vaccine effect that recruits cytotoxic T lymphocytes and elicits systemic antitumor immunity against local and distant tumors.

## 2 Results and Discussion

### 2.1 Preparation and Characterization of EP

We developed a bacterial surface modification strategy using tannic acid (TA) [38, 39], a natural polyphenol rich in catechol groups. These groups coordinate strongly with metal ions and form reversible boronic ester bonds with phenylboronic acid derivatives (Figure 2a). This strategy allowed us to covalently conjugate boronophenylalanine (BPA) to the bacterial surface through pH‐sensitive boronic ester linkages. We chose the probiotic *Escherichia coli* Nissle 1917 as our microbial chassis because of its well‐documented safety in humans and inherent tumor‐targeting properties [40, 41].

**FIGURE 2:**
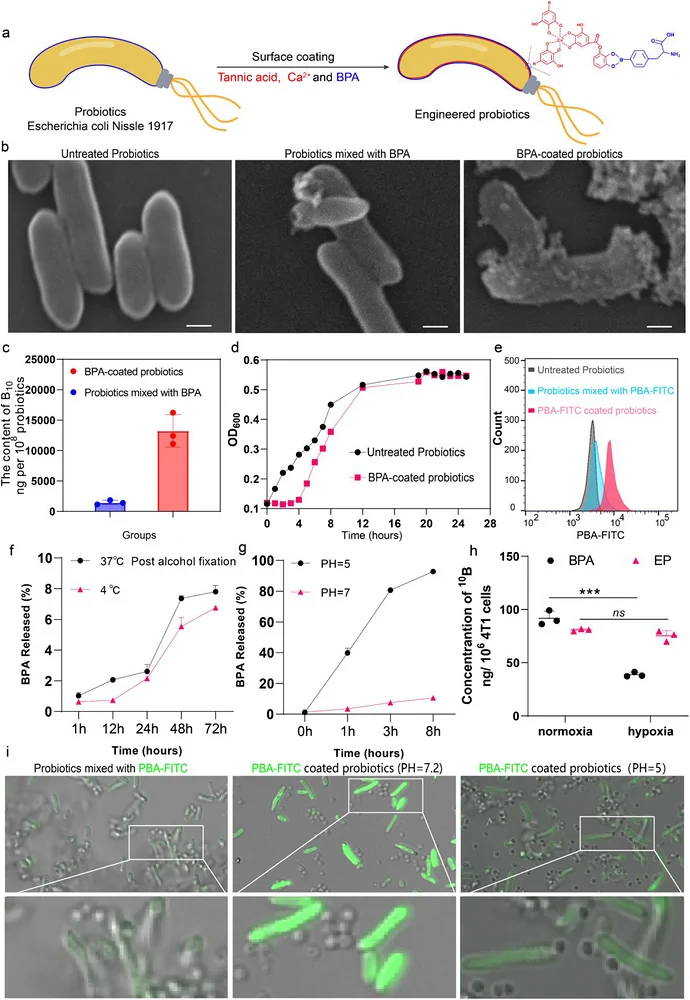
Characterization of the engineered probiotics. (a) Schematic illustration of the bacterial surface modification strategy. Tannic acid (TA), rich in catechol groups, forms a coating on the probiotic *Escherichia coli* Nissle 1917, enabling subsequent covalent conjugation of BPA via pH‐sensitive boronic ester bonds. (b) Representative scanning electron microscopy (SEM) images showing the continuous layer formed by TA coating on the bacterial surface. (c) Quantitative analysis of boron loading capacity (µg per 10^8^ bacteria) determined by inductively coupled plasma mass spectrometry (ICP‐MS). Data are presented as mean ± s.d. (*n* = 3 independent samples). (d) Bacterial growth curves of unmodified and BPA‐functionalized probiotics. (e) Flow cytometry analysis of fluorescence intensity from bacteria subjected to simple mixing with FITC‐labeled phenylboronic acid (PBA) vs. those with the TA‐coated, conjugated approach (*n* = 3). (f) Stability Assays of EP. Data are presented as mean ± s.d. (*n* = 3). (g) Acid‐Responsive Release of EP. Data are presented as mean ± s.d. (*n* = 3). (h) Boron content in EP‐co‐incubated tumor cells. Data are presented as mean ± s.d. (*n* = 3). The data were analyzed by one way ANOVA with Holm–Šídák multiple‐comparisons test. nsp >0.05, *p < 0.05, **p < 0.01, ***p < 0.001, and ****p < 0.0001. (i) Confocal microscopy images of bacteria with the FITC‐ PBA conjugate under neutral (pH 7.4) and acidic (pH 6.0) conditions, demonstrating the pH‐responsive release of the surface coating (*n* = 3).

Initial attempts to adsorb BPA directly onto unmodified bacteria were unsuccessful, likely due to the compound's inherent hydrophobicity (Figure 2b). In contrast, scanning electron microscopy (SEM) showed that TA coating formed a continuous layer over the bacterial surface (Figure 2b). Quantitative analysis by inductively coupled plasma mass spectrometry (ICP‐MS) confirmed efficient boron loading onto TA‐coated bacteria, reaching 15 µg per 10^8^ cells (Figure 2c). This loading capacity is substantially higher than that achieved by passive adsorption and provides sufficient boron for subsequent neutron capture reactions. Growth kinetics experiments showed that BPA‐functionalized probiotics had a slightly longer lag phase initially, but then grew rapidly to reach final densities similar to unmodified controls (Figure 2d). This indicates that although the coating process initially stresses bacterial metabolism, the cells adapt and retain full replicative capacity. Flow cytometry using FITC‐conjugated phenylboronic acid (PBA) showed much higher fluorescence intensity on coated bacteria than on bacteria treated by simple mixing (Figure 2e).

To assess the stability of coated EP, bacteria were killed and fixed with ethanol prior to storage stability evaluation. After 72 h of storage at room temperature, only ∼8% of the loaded BPA was released into the supernatant. A comparable release profile was observed at 4°C, with merely ∼7% BPA leakage over the same period (Figure 2f). The polyphenol‐based surface coating confers pH‐responsive release behavior, which aligns with the acidic microenvironment characteristic of most solid tumors. Accordingly, incubation of coated EP in pH 5.0 buffer resulted in approximately 80% of the loaded BPA being released into the supernatant within 3 h (Figure 2g). We further co‐cultured tumor cells with either coated EP or the conventional BPA‐sorbitol formulation. Under normoxic conditions, intracellular boron accumulation in tumor cells was comparable between the two groups. Intriguingly, the EP group exhibited higher intracellular BPA levels under hypoxic conditions (Figure 2h and Figure S1), which may be ascribed to upregulated macropinocytosis in tumor cells under hypoxic stress.

Confocal microscopy further confirmed uniform fluorescence distribution across the surface of coated bacteria (Figure 2i). Consistent with the known acid lability of boronic ester bonds, incubation of functionalized bacteria in pH 6.0 buffer for 30 min resulted in a marked decrease in fluorescence (Figure 2i). This pH‐responsive release profile is highly beneficial for tumor targeting [42], as the TME is characteristically acidic, allowing preferential release of PBA in tumor tissue for subsequent cellular uptake. This differential release between physiological and acidic pH should enhance therapeutic specificity by minimizing systemic exposure.

### 2.2 Engineered Probiotics with Radiation‐Triggered Gene Expression

Having established efficient BPA functionalization of the bacterial surface, we engineered radiation‐responsive genetic circuits. Gamma irradiation is known to specifically induce RecA expression [43], so we used the recA promoter to drive expression of the green fluorescent protein (GFP) reporter (Figure 3a). Following low‐dose neutron irradiation, flow cytometry showed no significant increase in fluorescence in non‐boron‐loaded bacteria (Group P) relative to non‐irradiated controls. In contrast, boron‐loaded engineered probiotics (Group EP) showed markedly stronger GFP fluorescence than both Group P and untreated controls (Figure 3b). Fluorescence imaging of liquid cultures and solid agar plates consistently confirmed higher signal intensity in irradiated EP groups compared with all controls (Figure 3c).

**FIGURE 3:**
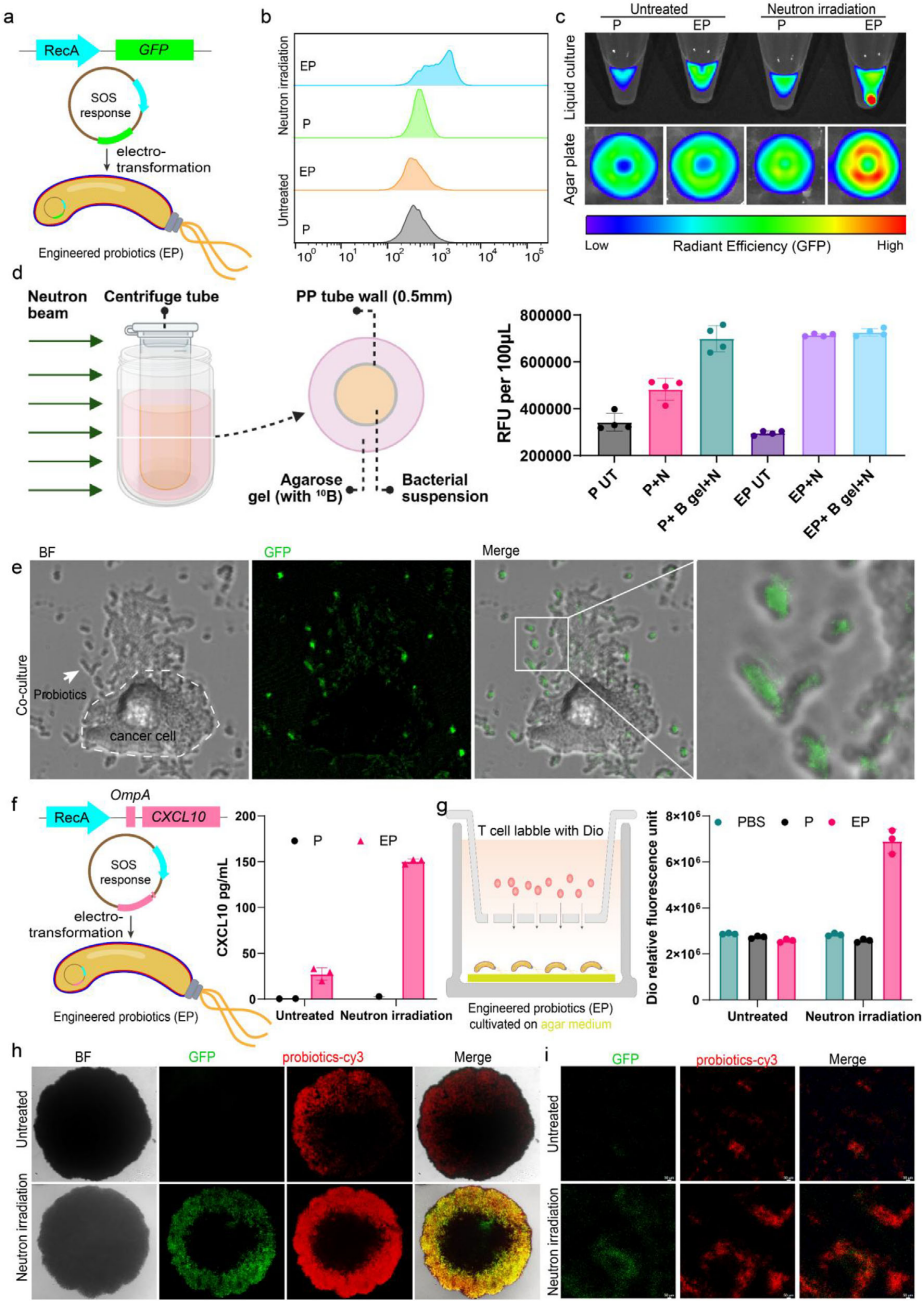
Engineered probiotics with radiation‐triggered gene expression. (a) Schematic of the synthetic genetic circuit utilizing the radiation‐inducible RecA promoter to drive expression of green fluorescent protein (GFP) or the chemokine CXCL10. (b) Flow cytometry analysis of GFP fluorescence in bacterial populations following neutron irradiation. P: non‐boron‐loaded bacteria; EP: boron‐loaded engineered probiotics. (c) Fluorescence images of liquid cultures (top) and solid agar plates (bottom) from different treatment groups after irradiation. (d) Spatial separation assay for irradiation‐responsive expression. Data are presented as mean ± s.d. (*n* = 4 independent experiments). (e) Confocal microscopy images showing GFP expression (green) in bacteria (red) specifically proximal to tumor cells (blue) after neutron irradiation, demonstrating spatial specificity of BNCR sensing. (f) left: Design of the CXCL10 secretion circuit featuring an OmpA signal peptide and a thrombin‐cleavable linker. Right: ELISA quantification of CXCL10 secretion in culture supernatants from irradiated groups. Data are presented as mean ± s.d. (*n* = 3 independent experiments). (g) T‐cell migration assay. Left: Schematic of the Transwell setup. Right: Quantification of migrated CD3^+^ T cells toward conditioned media from different bacterial groups with or without neutron irradiation. Data are presented as mean ± s.d. (*n* = 3 independent experiments). (h) Confocal microscopy images showing GFP expression (green) in Cy3‐labeled bacteria (red) infiltrating the periphery of tumor spheroids, with DAPI staining (blue) indicating tumor cells. (i) Experimental timeline of the 4T1 murine tumor model. Fluorescence imaging of tumor tissues. Representative images showing GFP signal in tumors from different treatment groups.

We next examined the spatial specificity of boron neutron capture reaction (BNCR) sensing. To directly verify whether non‐dominant radiation doses during BNCT are sufficient to activate the RecA promoter, we have supplemented a dedicated spatial separation experiment. Engineered bacteria carrying a RecA promoter‐driven GFP reporter were sealed inside a 0.5 mm‐thick polypropylene (PP) centrifuge tube, which was embedded in agarose gel enriched with ^10^B‐boric acid. Given the short tissue range (only several micrometers) of α‐particles and ^7^Li nuclei, the 0.5 mm PP wall completely blocks these high‐LET dominant products of the ^10^B(n,α)^7^Li nuclear reaction, while allowing non‐dominant radiation components—including neutrons, secondary γ‐rays, and tissue recoil nuclei—to reach the bacteria inside the tube (Figure 3d). The results show that significant GFP fluorescence was detected in bacteria inside the tube (with no direct contact with boron agents) after neutron irradiation, providing direct evidence that non‐dominant radiation doses from BNCT are sufficient to activate the bacterial RecA promoter. We also observed a moderate increase in fluorescence intensity in the boron‐free control group compared with the unirradiated group, indicating that neutrons themselves induce a degree of genotoxic stress (Figure 3d). These results demonstrate that boron‐loaded bacteria specifically amplify radiation signals through neutron capture reactions, producing a measurable biological response. Tumor cells were pre‐incubated with BPA, co‐cultured with non‐boron‐loaded bacteria, and then exposed to neutron irradiation. Confocal microscopy showed GFP expression exclusively in bacteria adjacent to tumor cells (Figure 3e), confirming that the RecA promoter effectively detects localized BNCR events. This spatial precision provides a mechanism to restrict therapeutic payload activation to tumor sites in future applications.

While BNCT effectively ablates bulk tumor tissue, residual cells often remain and cause disease recurrence. Concurrently, therapy‐induced tumor cell death releases large quantities of tumor antigens. We hypothesized that enhancing immune recognition of these antigens would promote elimination of residual cells and prevent relapse. After systematic evaluation of multiple immunomodulatory candidates, we selected CXCL10 as the therapeutic payload for this strategy. As a well‐characterized CXCR3‐binding chemokine, CXCL10 drives recruitment of CD8^+^ cytotoxic T lymphocytes, NK cells and Th1 cells into tumor tissues, promotes dendritic cell maturation and antigen cross‐presentation, and is clinically associated with favorable prognosis and improved immunotherapy response. This chemokine is particularly well suited for combination with BNCT. BNCT elicits robust immunogenic cell death to release tumor antigens, but full development of antitumor immunity depends on efficient trafficking of effector T cells to the tumor microenvironment; localized CXCL10 expression directly bridges this rate‐limiting step. In addition, the immune‐sparing feature of BNCT ensures that recruited immune cells remain functionally competent, further amplifying the therapeutic effect. Compared with pleiotropic proinflammatory cytokines such as IL‐2, IFN‐γ, and TNF‐α, which carry high risks of systemic toxicity and off‐target immunosuppression, CXCL10 exerts localized chemotactic effects with a more favorable safety profile, making it optimal for sustained intratumoral delivery via engineered live bacteria. To test this, we engineered bacteria to secrete the T‐cell chemoattractant CXCL10. We constructed a secretion cassette in which CXCL10 was fused to the OmpA signal peptide via a thrombin‐cleavable linker (Figure 3f). This design takes advantage of previous observations that bacteria induce tumor hemorrhage and subsequent coagulation [44, 45], which would trigger localized chemokine release through thrombin‐mediated linker cleavage.

Enzyme‐linked immunosorbent assay (ELISA) of bacterial culture supernatants confirmed significantly higher CXCL10 secretion in irradiated EP groups compared with irradiated non‐boron‐loaded Group P (Figure 3f). To assess the bioactivity of secreted CXCL10, we performed Transwell migration assays using CD3^+^ T cells enriched from expanded mouse splenocytes. EP bacteria grown on solid agar were irradiated with neutrons before adding T cells. Irradiated EP groups showed substantially greater T‐cell migration than all controls (Figure 3g), confirming that the secreted chemokine is functional. The level of T‐cell recruitment indicates that bacterially produced CXCL10 retains full chemotactic activity and can effectively modulate immune cell trafficking in tumors.

Encouraged by these in vitro findings, we evaluated the system in tumor models. In 3D tumor spheroids co‐cultured with EP bacteria, we observed strong GFP expression specifically in bacteria that had infiltrated the periphery of irradiated spheroids, with no such signal in non‐irradiated controls (Figure 3h). Notably, neutron irradiation also increased the depth of bacterial infiltration into spheroids. We extended these studies to a 4T1 murine mammary carcinoma model, where EP bacteria within tumor tissue showed significantly stronger GFP signals after neutron irradiation compared with controls (Figure 3i). These results demonstrate that our engineered probiotics have dual therapeutic functions: they act as precise BNCR sensors via RecA‐mediated genetic circuits, and as targeted immune modulators through in situ secretion of functional T‐cell chemokines. The consistent activation pattern across all model systems confirms that the radiation‐responsive circuitry operates reliably in complex tissue environments.

### 2.3 Engineered Probiotics Synergize with Boron Neutron Capture Therapy

Having validated the functionality of our EP strains, we investigated their ability to enhance BNCT efficacy. Engineered probiotics were co‐cultured with tumor cells for 3 h, followed by neutron irradiation and a further 12 h incubation before apoptosis analysis. Flow cytometry showed marked apoptosis (Annexin V^+^/PI^+^ populations) in EP groups, whereas negligible cell death occurred in groups treated with non‐boron‐loaded bacteria or equivalent doses of free BPA (Figure 4a). This enhanced cytotoxicity may be attributed to bacterial colonization of the abundant apoptotic cells generated during BNCT, which may inhibit cellular repair mechanisms that would otherwise limit treatment efficacy. This close proximity to damaged cells also amplifies the therapeutic effect locally through multiple bystander pathways.

**FIGURE 4:**
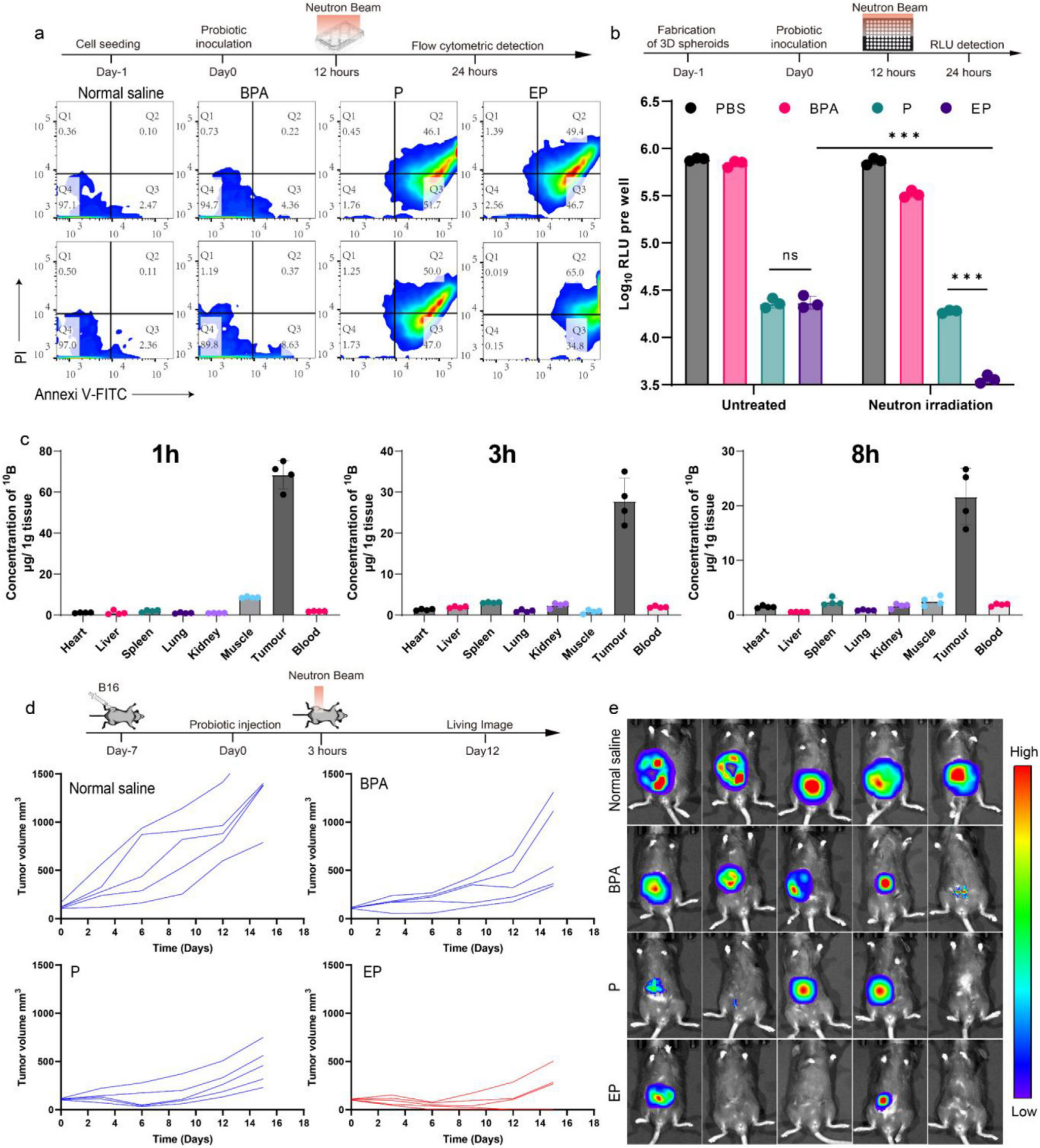
Engineered probiotics synergizes with boron neutron capture therapy. (a) Flow cytometry analysis of tumor cell apoptosis after various treatments. (b) Viability assessment of 3D tumor spheroids post‐treatment. Quantification of bioluminescence intensity. Data are presented as mean ± s.d. (*n* = 3 spheroids per group). (c) Boron concentration at various time points following intratumoral EP administration. Data are presented as mean ± s.d. (*n* = 4 independent experiments). (d) Experimental timeline of the B16 murine tumor model. Bacteria were intratumorally injected, followed by neutron irradiation at the indicated time point. (e) Bioluminescence images of B16 tumor‐bearing mice from different groups.

We next assessed therapeutic efficacy in 3D tumor spheroids (Figure 4b). Notably, luminescence signals in EP groups were almost completely abolished, while strong signals remained in PBS and free BPA control groups (Figure 4b). Consistent with this, EP treatment resulted in significantly lower tumor cell viability post‐irradiation compared with non‐boron‐loaded bacteria. This near‐complete eradication of viable tumor cells indicates a synergistic interaction between BNCT and bacterial effects, rather than merely additive cytotoxicity.

We then extended our studies to in vivo subcutaneous B16 melanoma models. To identify a suitable delivery route, we first evaluated systemic administration. Notably, the BPA coating density on the bacterial surface cannot be increased indefinitely: excessive surface modification substantially impairs bacterial viability, colonization capacity, and intrinsic biological functions. Even at the maximally tolerated dose of coated bacteria delivered via systemic injection, intratumoral boron concentrations remained well below the 20 µg/g threshold required for effective BNCT (Figure S2).

Accordingly, we adopted intratumoral administration in the present study, with a primary focus on investigating the abscopal effect and metastatic suppression elicited by BNCT following local delivery. From a clinical perspective, image‐guided local injection and perfusion are feasible for most solid tumor types.

To define the optimal time window for neutron irradiation, we collected major organs, blood, tumor tissue, and peritumoral muscle from tumor‐bearing mice at serial time points after injection, and quantified boron concentrations across all samples (Figure 4c). At 1 h post‐injection, detectable boron was almost exclusively restricted to the tumor and immediately adjacent muscle, with no significant accumulation observed in any other major organs examined. By 3 h post‐injection, boron levels in peritumoral muscle had declined markedly, while intratumoral boron concentrations remained robust. At this time point, both the tumor‐to‐normal tissue (T/N) ratio and tumor‐to‐blood (T/B) ratio exceeded 3, meeting the widely accepted threshold for a safe and effective BNCT therapeutic window. This favorable biodistribution profile constitutes the core rationale for selecting 3 h post‐injection as the neutron irradiation timing in all therapeutic experiments. Mice received intratumoral injections of 1 × 10^6^ CFU bacteria, followed by neutron irradiation using a custom‐built device that eliminates the need for animal anesthesia. EP treatment significantly inhibited tumor growth compared with mice receiving non‐boron‐loaded bacteria or equivalent doses of free BPA (Figure 4d). Complete tumor regression was observed in 40% of EP‐treated mice (Figure 4d,e).

These results establish our EP platform as an effective strategy to enhance BNCT efficacy. The superior performance of EP compared with free BPA, despite identical boron doses, underscores the critical role of delivery strategy and indicates that bacterial localization improves boron utilization efficiency.

### 2.4 Engineered Bacteria‐Mediated Radioimmunotherapy

The promising results with GFP‐expressing EP led us to evaluate CXCL10‐secreting EP for radioimmunotherapy. In the B16 tumor model, mice received intratumoral injections of 5 × 10^6^ CFU EP followed by neutron irradiation 12 h later. While increasing the bacterial dose did not significantly improve efficacy compared with 1 × 10^6^ CFU (Figure 4d), CXCL10‐secreting EP nonetheless markedly inhibited tumor growth after irradiation relative to saline, BPA‐only, and non‐boron‐loaded bacterial controls (Figure 5a–e), confirming synergistic activity between BNCT and the engineered probiotics. Notably, in EP‐treated groups, only one of six tumors regrew after day 8, and its progression was much more slowly than tumors in all other groups (Figure 5d,e), indicating that CXCL10 secretion contributes to suppression of recurrence.

**FIGURE 5:**
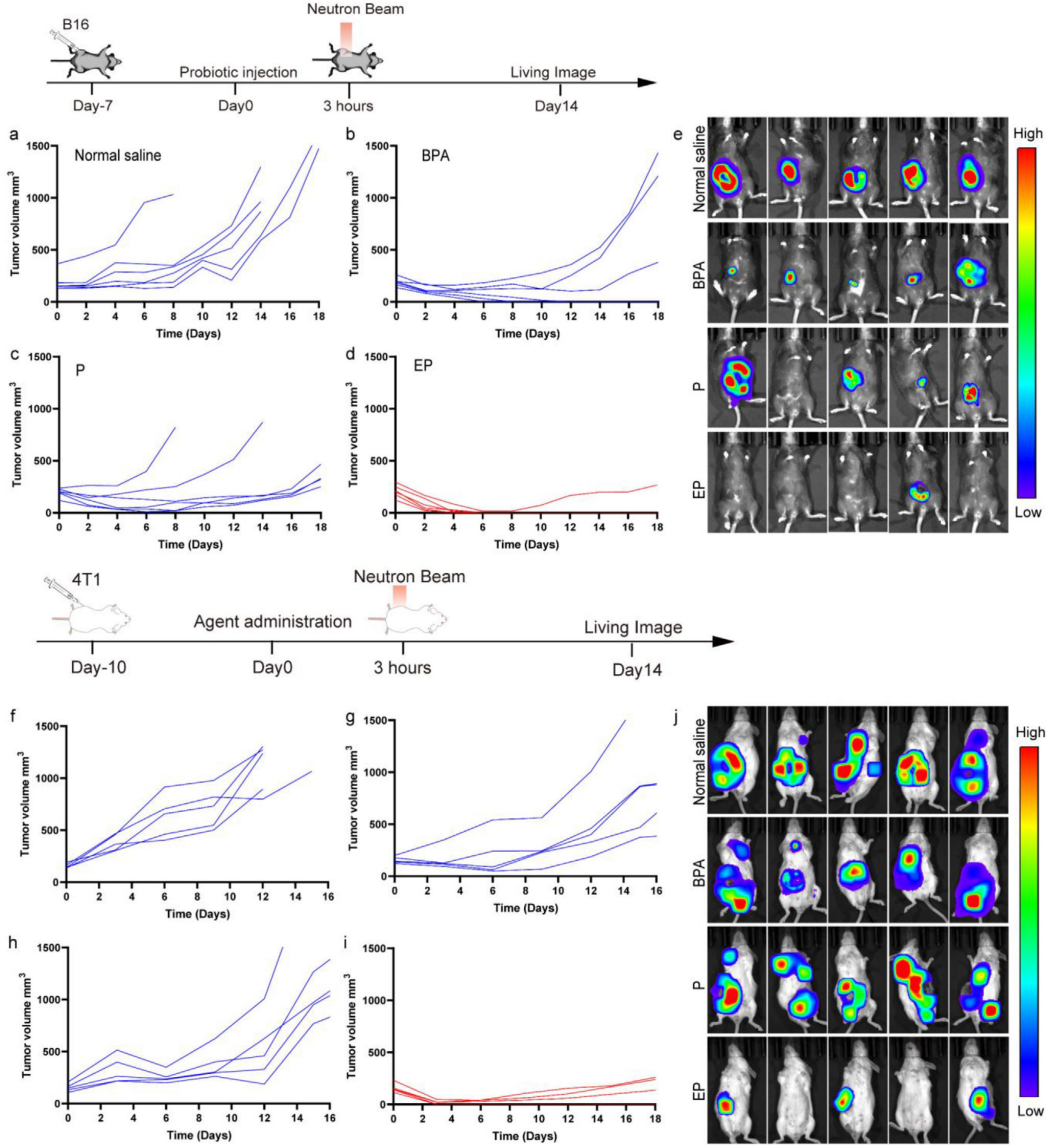
Engineered probiotics‐mediated radioimmunotherapy. (a–e) Evaluating CXCL10‐secreting EP in the B16 melanoma model. (a–d) Tumor growth curves of mice receiving different treatments. Each curve represents an individual mouse. (e) bioluminescence images of B16 tumor‐bearing mice from different treatment groups. (f–j) Evaluating CXCL10‐secreting EP in the 4T1 breast cancer model. (f–i) Tumor growth curves of mice receiving different treatments. Each curve represents an individual mouse. (j) Bioluminescence images of 4T1 tumor‐bearing mice from different treatment groups.

As B16 melanoma is a well‐characterized immunologically “hot” tumor model, we further tested our approach in the immunologically “cold” 4T1 breast cancer model (Figure 5f–j). Strikingly, neither bacterial administration alone (Figure 5g) nor equivalent doses of free BPA (Figure 5h) produced any significant therapeutic effect in this model, likely due to the dense, hypoxic stroma characteristic of 4T1 tumors. In contrast, EP treatment significantly delayed tumor growth (Figure 5i) and resulted in complete regression in 40% of mice (Figure 5j).

These collective results demonstrate that our EP platform converts a key limitation of conventional BNCT into a therapeutic advantage. It establishes a self‐reinforcing cycle in which BNCT‐induced tumor damage enhances bacterial colonization of hypoxic niches, while the resulting γ‐rays activate our genetic circuits to drive localized CXCL10 expression, ultimately amplifying anti‐tumor immunity. The efficacy observed in both immunologically “hot” and “cold” tumor models suggests that the platform functions effectively across diverse tumor microenvironments, likely via multiple complementary mechanisms.

### 2.5 BNCT Promotes the Proliferation of Probiotics Within Tumors

We next investigated whether BNCT reciprocally enhances bacterial tumor colonization. 3D tumor spheroids were inoculated with Cy3‐labeled bacteria, and neutron irradiation was applied after 1 h of co‐culture. Following a further 5 h incubation, EP bacteria had fully penetrated the spheroid cores, whereas non‐boron‐loaded (P) bacteria showed only limited infiltration (Figure 6a). Irradiated spheroids co‐cultured with EP also appeared more translucent, consistent with extensive tissue damage. Colony‐forming unit (CFU) enumeration after PBS washing confirmed significantly higher bacterial retention in irradiated EP groups (Figure S3).

**FIGURE 6:**
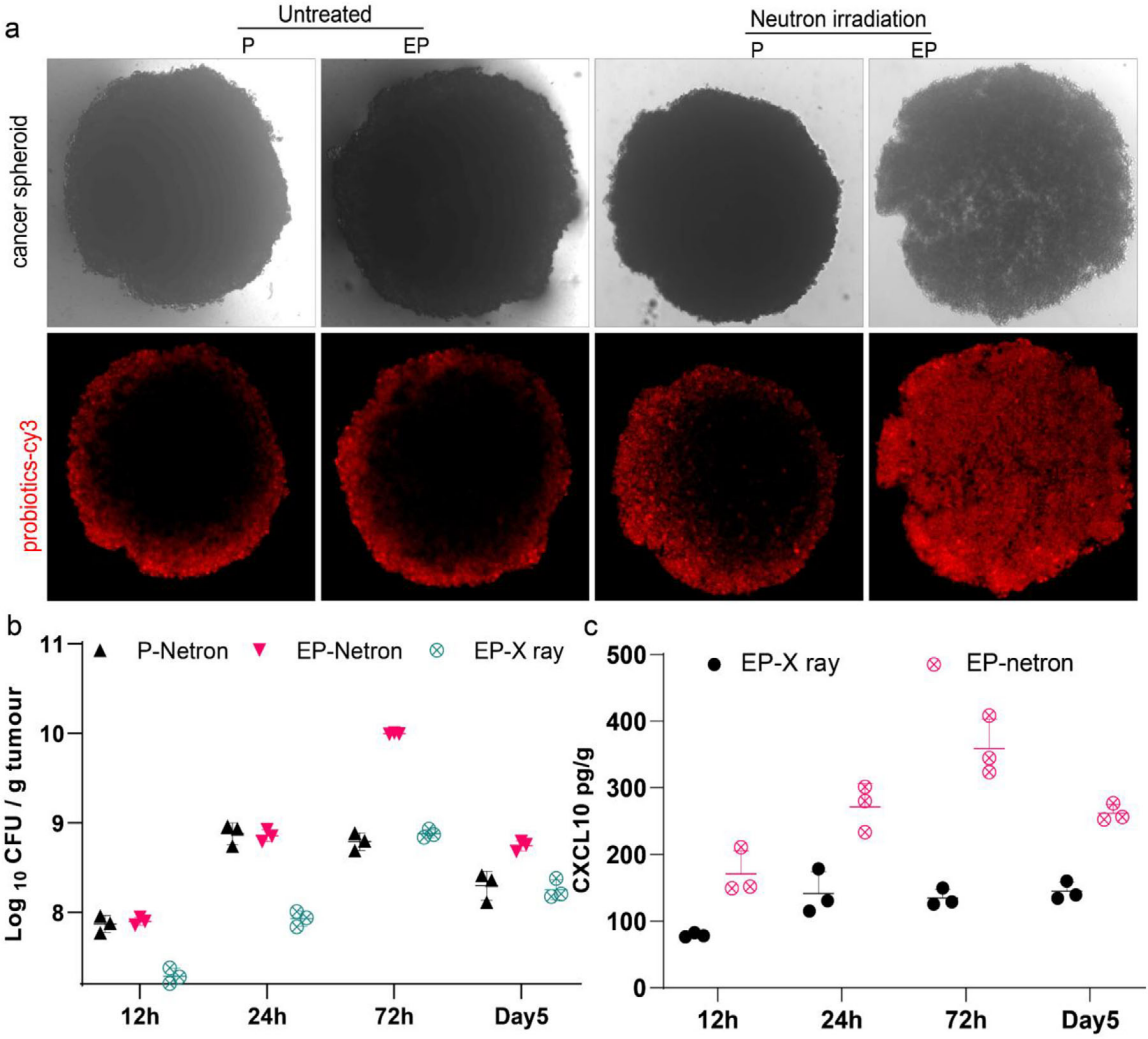
BNCT promotes the proliferation of probiotics within tumors. (a) 3D tumor spheroid infiltration assay. Confocal microscopy images showing Cy3‐labeled bacteria (red) within tumor spheroids at 5 h post‐irradiation. (b) Bacterial colonization dynamics in 4T1 tumor‐bearing mice following neutron and x‐ray irradiation. CFU counts in tumor tissues were measured at the indicated time points post‐irradiation. Data are presented as mean ± s.d. (*n* = 3 mice per group). (c) Tumor tissue CXCL10 levels across time points. Data are presented as mean ± s.d. (*n* = 3 mice per group).

To validate these in vitro observations in a physiological context and directly compare the effects of different radiation modalities on bacterial survival, we performed parallel experiments in 4T1 tumor‐bearing mice. At 12 h post‐irradiation, bacterial burdens in both neutron‐irradiated P and EP groups were significantly higher than those in x‐ray‐irradiated mice (Figure 6b). This critical difference arises because x‐rays induce non‐specific DNA damage across all cells within the radiation field, including intratumoral bacteria. Neutron irradiation therefore does not indiscriminately eliminate intratumoral bacteria, preserving the microbial population required for subsequent therapeutic effects. It should be noted that, due to the inherent biophysical differences between high‐LET boron neutron capture therapy and low‐LET x‐rays, strict dose matching between neutron and x‐ray irradiation was not performed. We cannot completely exclude the possibility that dose discrepancies may have confounded the observed differences in bacterial survival. However, this limitation does not alter the central conclusion of our study: that BNCT preserves intratumoral bacterial viability, a phenomenon dictated by the spatial characteristic that the boron neutron capture reaction is confined exclusively to boron‐loaded cells.

By 24 h post‐irradiation, bacterial numbers had increased markedly in both neutron‐ and x‐ray‐irradiated groups compared to non‐irradiated controls. This universal increase reflects the release of intracellular nutrients from radiation‐induced apoptotic tumor cells, which provides a growth advantage for facultative anaerobic bacteria that preferentially colonize necrotic tumor regions. Notably, a significant divergence in bacterial burdens emerged by 72 h post‐irradiation: EP‐treated mice had substantially higher bacterial loads than P‐treated mice receiving the same neutron dose (Figure 6b). This difference is directly attributable to the enhanced BNCT efficacy in EP groups, which generates a larger pool of necrotic tumor tissue and nutrients to support bacterial proliferation, initiating the positive feedback loop described earlier. By day 5, bacterial numbers in all groups had declined from their 72 h peaks, consistent with the onset of adaptive immune responses that clear intratumoral bacteria. Importantly, EP groups still maintained the highest bacterial burdens among the three treatment groups at this time point, indicating that the therapeutic microbial population persists long enough to exert its full immunomodulatory effects before being eliminated (Figure 6b). This self‐limiting colonization profile is a key safety feature of the platform, preventing systemic bacterial dissemination. To evaluate the impact of BNCT on intratumoral CXCL10 production, we performed time‐course measurements of CXCL10 levels in tumor tissue following treatment. Compared with both the untreated control group and the conventional x‐ray irradiation group, the BNCT treatment group exhibited significantly higher intratumoral CXCL10 concentrations at all assessed time points (Figure 6c). This finding confirms that BNCT is able to induce robust upregulation of CXCL10 within the tumor microenvironment, providing experimental support for this key node of our proposed mechanism.

This discrepancy in bacterial survival is further compounded by divergent effects of the two radiation modalities on intratumoral immune populations, which together account for the substantial efficacy gap between BNCT–bacteria and X‑ray–bacteria combination regimens. Conventional X‑ray radiotherapy delivers uniform low‑LET energy across the entire radiation field, causing non‑specific DNA damage not only to tumor cells and bacteria, but also to tumor‑infiltrating immune cells including cytotoxic T lymphocytes and dendritic cells. This direct immune cell ablation, combined with radiation‑induced upregulation of immunosuppressive pathways (e.g., PD‑L1 upregulation and MDSC recruitment), creates a dual bottleneck for bacteria‑mediated immunotherapy: it reduces the viable bacterial population available for immune stimulation, and simultaneously impairs the downstream effector immune cells required to translate bacterial adjuvant activity into tumor rejection. In contrast, the spatially confined high‑LET energy deposition of BNCT selectively targets boron‑loaded tumor cells while largely sparing intratumoral bacteria and immune cells. This preservation of both the bacterial delivery platform and the host immune compartment enables effective coupling of BNCT‑induced immunogenic cell death and bacteria‑driven immune modulation. The retained probiotic population sustains CXCL10 secretion and innate immune adjuvant effects, while preserved immune cells remain competent to respond to tumor antigens and execute cytotoxic functions, forming a self‑reinforcing antitumor immune cycle.

### 2.6 Engineered Probiotic‐Mediated BNCT Induces Abscopal Effects

We further evaluated the abscopal vaccine‐like activity of EP‐mediated radioimmunotherapy using bilateral tumor models (Figure 7a–c). We first tested this in the highly immunogenic B16 melanoma model, where EP treatment not only potently suppressed primary irradiated tumors (Figure 7a,c) but also significantly inhibited growth of distant tumors (Figure 7b,c). Remarkably, this abscopal effect was also observed in the poorly immunogenic “cold” 4T1 breast cancer model, which is notoriously resistant to conventional immunotherapies (Figure 7d–f). EP treatment elicited robust therapeutic responses in both primary (Figure 7d,f) and distant tumors (Figure 7e,f), an effect not observed with equivalent doses of free BPA (Figure 7d–f).

**FIGURE 7:**
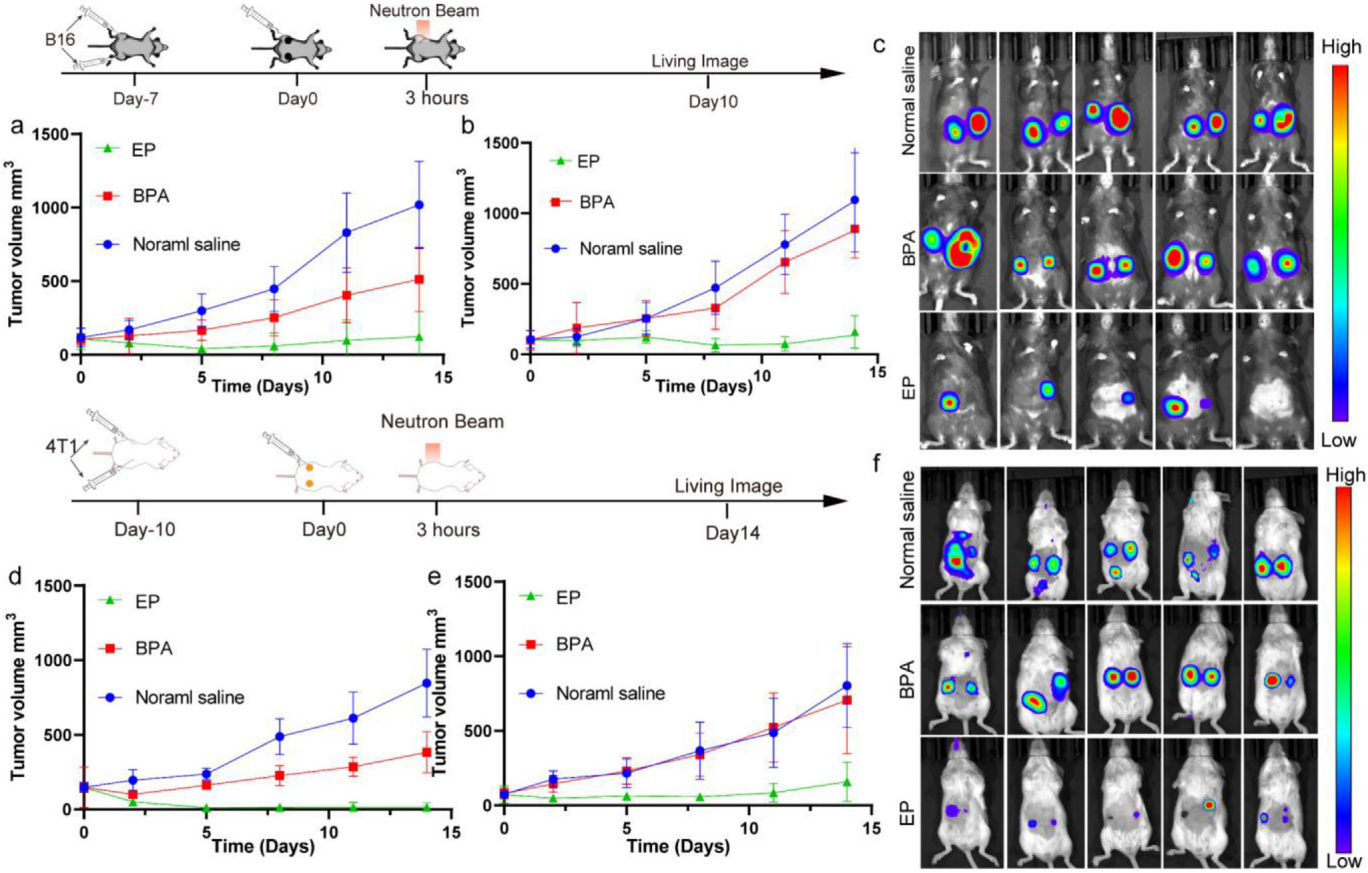
Engineered probiotic‐mediated BNCT induces abscopal effects. (a) Growth curves of primary irradiated tumors. Data are presented as mean ± s.d. (*n* = 5 mice per group). (b) Growth curves of distant non‐irradiated tumors. Data are presented as mean ± s.d. (*n* = 5 mice per group). (c) Bioluminescence images showing systemic tumor control in the bilateral model. (d) Growth curves of primary irradiated tumors. Data are presented as mean ± s.d. (*n* = 5 mice per group). (e) Growth curves of distant non‐irradiated tumors. Data are presented as mean ± s.d. (*n* = 5 mice per group). (f) Bioluminescence images showing systemic tumor control in the bilateral model.

This abscopal activity indicates that EP‐based therapy converts treated tumors into effective in situ vaccination sites. BNCT induces robust immunogenic cell death, releasing a broad repertoire of tumor‐associated antigens that are taken up and cross‐presented by dendritic cells and other antigen‐presenting cells. Concurrently, EP‐secreted CXCL10 recruits circulating cytotoxic T lymphocytes to the tumor microenvironment, where they are primed against the presented antigens. These activated tumor‐specific T cells then enter the systemic circulation and traffic to distant sites to eliminate metastatic tumor cells. The induction of durable systemic antitumor immunity has critical clinical implications for the management of metastatic disease, as it enables the platform to simultaneously target primary tumors and occult micrometastases.

### 2.7 Engineered Probiotics‐Mediated BNCT Enhances Immune Surveillance

Immune escape‐driven metastasis remains the leading cause of mortality in triple‐negative breast cancer patients. Building on our demonstration that EP mediates effective radioimmunotherapy, we evaluated its potential as a neoadjuvant therapy to prevent postoperative metastasis. We established a surgical resection model using 4T1 triple‐negative breast cancer, where primary tumors were surgically removed 14 days after BNCT, and metastatic progression was monitored by bioluminescence imaging of major organs until day 32 (Figure 8a).

**FIGURE 8:**
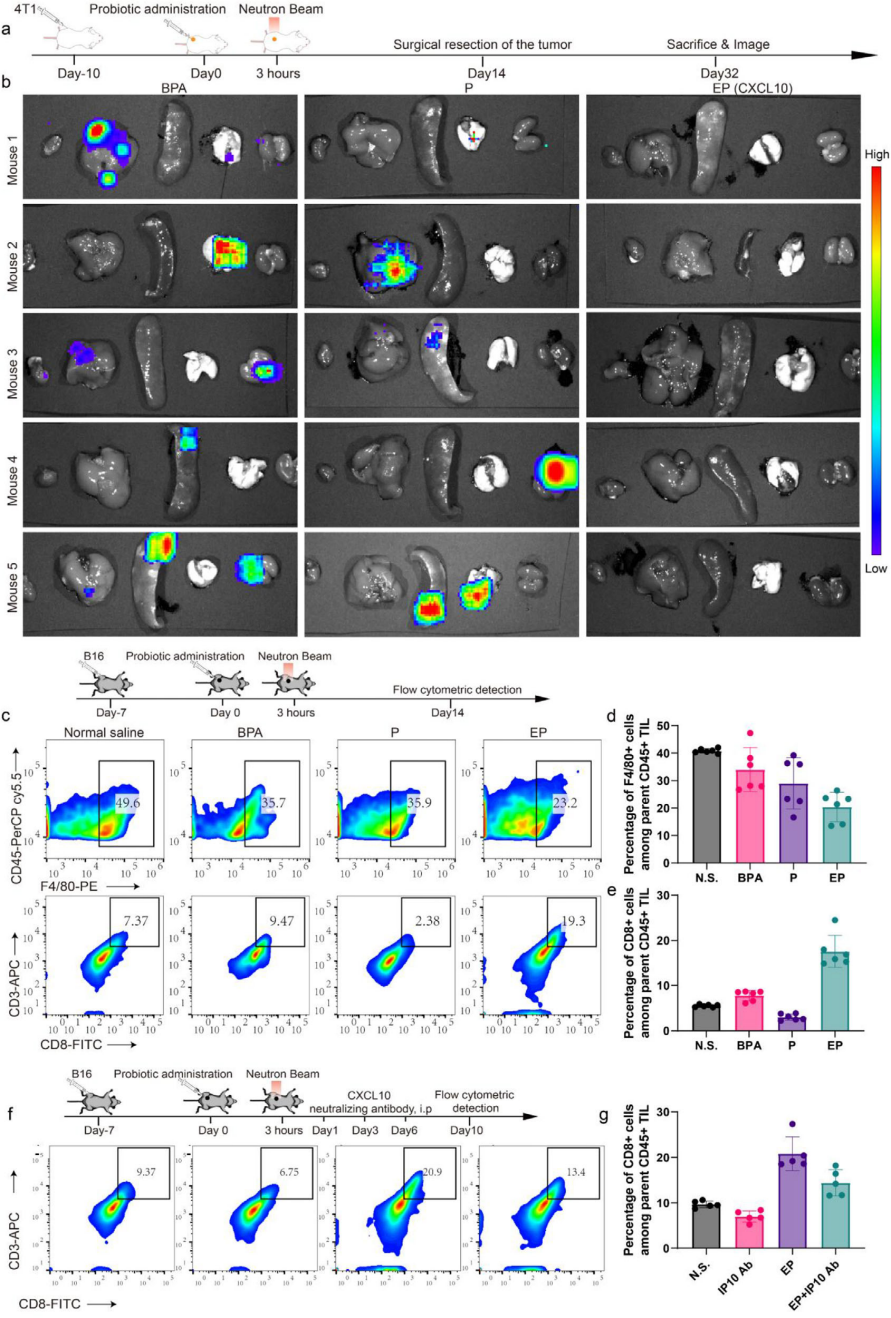
Engineered probiotics‐mediated BNCT enhances immune surveillance. (a) Experimental timeline of the spontaneous metastasis model. (b) Bioluminescence imaging of major organs (heart, liver, spleen, lung, and kidney) collected on day 32, showing metastatic burden across treatment groups. (c) Representative flow cytometry plots showing immune cell populations in tumor tissues. (d) Quantification of macrophages (F4/80^+^CD11b^+^) among CD45^+^ cells. (e) Percentages of CD8^+^ cytotoxic T cells among CD45^+^ cells. Data are presented as mean ± s.d. (*n* = 6 mice per group). (f–g) umor‐infiltrating CD8^+^ T cells after CXCL10 antibody neutralization. Data are presented as mean ± s.d. (*n* = 5 mice per group).

Strikingly, no detectable metastatic foci were observed in any EP‐treated mice, whereas extensive metastases developed in all mice receiving either free BPA or non‐boron‐loaded bacteria (P) (Figure 8b). To investigate the immune mechanisms underlying this complete protection against metastasis, we performed flow cytometric analysis of primary tumor tissues harvested at the time of resection (day 14). EP‐treated tumors showed significantly reduced proportions of tumor‐associated macrophages (TAMs) (Figure 8c,d) and markedly increased frequencies of CD3^+^ total T cells and CD8^+^ cytotoxic T lymphocytes (CTLs) compared with all control groups (Figure 8c,e). The enrichment of functional CD8^+^ CTLs in EP‐treated tumors is likely the primary driver of the complete metastasis suppression observed, as these cells are critical for eliminating circulating tumor cells and micrometastatic lesions. These immune profiling data align with the mechanistic rationale that BNCT preserves intratumoral immune cell viability relative to conventional X‑ray radiotherapy, allowing engineered probiotics to fully exert their immunomodulatory effects and drive robust T cell‑mediated immune surveillance against metastatic dissemination.

To provide additional supporting evidence for this mechanism, we employed a CXCL10 neutralizing antibody from BioXCell and performed in vivo neutralization assays. In these experiments, the CXCL10 neutralizing antibody was administered via intraperitoneal (i.p.) injection concurrently with our bacterial treatment regimen, and intratumoral CD8^+^ T cell infiltration was subsequently quantified by flow cytometry (Figure 8f–g). We found that CXCL10 neutralization significantly attenuated the increase in CD8^+^ T cell infiltration induced by EP treatment, indicating that the therapeutic benefit of EP bacteria is mediated, at least in part, by their secreted CXCL10.

The bidirectional immune remodeling—reduced TAM infiltration and expanded CD8^+^ T cell populations—arises from the synergistic action of BNCT and engineered probiotics. BNCT elicits robust immunogenic cell death that releases tumor antigens and damage‐associated molecular patterns to prime adaptive immunity, while the spatially confined energy deposition avoids non‐specific ablation of tumor‐infiltrating immune cells. Concurrently, radiation‐triggered CXCL10 secretion by engineered probiotics directly chemoattracts circulating CXCR3^+^ effector T cells into the tumor parenchyma, bridging the critical gap between antigen release and effector cell infiltration. Meanwhile, BNCT‐mediated tumor damage downregulates myeloid‐recruiting chemokines such as CCL2 and CSF‐1, and bacterial TLR stimulation suppresses the accumulation of immunosuppressive M2‐like macrophages, collectively dismantling the myeloid‐derived immunosuppressive barrier.

This immune profile shift bears a direct causal relationship to the observed therapeutic efficacy. Diminished TAM populations remove a major source of inhibitory cytokines (IL‐10, TGF‐β) that otherwise blunt T cell cytotoxicity, while the enriched, functionally competent CD8^+^ CTLs mediate direct killing of residual tumor cells. Together, these effects not only sustain primary tumor growth control but also drive systemic antitumor immunity that underlies the abscopal effect and complete protection against postoperative metastasis.

From a broader immunological perspective, this combinatorial strategy resolves a long‐standing limitation of conventional radioimmunotherapy. Unlike photon radiotherapy, which induces immunogenic cell death but simultaneously impairs immune cell function and exacerbates immunosuppression, our BNCT–probiotic platform couples tumor ablation with coordinated immune microenvironment remodeling. This approach provides a generalizable strategy to convert immunologically “cold” tumors into T cell‐inflamed tumors, and establishes a robust immunological foundation for further combination with immune checkpoint blockade or other systemic immunotherapies.

## 3 Conclusion

Our engineered probiotic platform establishes a modular framework with broad extensibility. The radiation‐responsive genetic circuits are highly reconfigurable and can be adapted to express a wide range of therapeutic payloads—including cytokines, antibody fragments, co‐stimulatory ligands, and prodrug‐converting enzymes—under the control of localized BNCR‐generated the low‐level incidental radiation. Rational engineering of bacterial surface moieties offers opportunities to further enhance tumor targeting specificity, tissue penetration, and intratumoral niche colonization, while incorporation of inducible self‐limiting and biocontainment systems will accelerate clinical translation by improving safety and controllability.

Beyond intrinsic platform optimization, combining this system with established therapeutic modalities such as immune checkpoint inhibitors, adoptive cell therapies, or targeted small molecules could yield additional synergistic benefits. Further refinement of bacterial colonization dynamics, boron loading strategies, and radiation dosing parameters will enable EP‐based systems to offer a viable path toward spatially programmable, bacteria‐directed cancer therapies. These approaches hold particular promise for treating a wide range of malignancies, including immunologically “cold” tumors that remain refractory to conventional radioimmunotherapy.

## 4 Experimental Section

### 4.1 Materials

Escherichia coli Nissle 1917, was purchased from China general microbiological culture collection center (GMCC, China). Antibodies against CD86 (cat. no. E‐AB‐F0994D, lot. no. WV06H8V4R4689), CD80 (cat. no. E‐AB‐F0992E, lot. no. WV074PRRT7973), CD11c (cat. no. E‐AB‐F0991C, lot. no. WV05006Z01268), CD3 (cat. no. E‐AB‐F1013C, lot. no. WU09F4J08470), CD4 (cat. no. E‐AB‐F1097D, lot. no. WU0816RF5343), CD8a (cat. no. E‐AB‐F1104E, lot. no. WU07408V2842), F4/80 (cat. no. E‐AB‐F0995D, lot. no. WU074L626113), CD103 (cat. no. E‐AB‐F1090C, lot. no. WU0886F29396) and CD45(cat. no. E‐AB‐F1136J, lot. no. WU10P48N2054) were purchased from Asbio Technology, Inc.

### 4.2 Cell Lines

Both B16 and 4T1 cell lines were purchased from Procell Life Science & Technology (Wuhan). B16 cells were cultured in Gibco's Dulbecco's Modified Eagle's Medium (DMEM), supplemented with 10% Gibco fetal bovine serum (FBS), 100 U/mL penicillin, 100 µg/mL streptomycin, and 50 µg/mL gentamicin. The culture environment was set as a humidified incubator at 37°C with 5% carbon dioxide. 4T1 cells were cultured in Roswell Park Memorial Institute 1640 medium (RPMI 1640), and their culture conditions were consistent with those of B16 cells except for the type of medium. During the culture period, the cell confluence needed to be controlled below 60%, and early passage cells (passages 4–9) were selected for the experiments.

### 4.3 Animals

C57BL/6 mice weighing 18–20 grams were acquired from GemPharmatech. Throughout the study, the animals were monitored on a daily basis to detect any clinically significant abnormalities. Euthanasia would be conducted on the mice if they became moribund as a result of therapeutic toxicity, developed severe ulceration around the subcutaneous tumor, or experienced a 20% reduction in body weight relative to the pre‐study baseline. All animal experiments were carried out in strict accordance with the protocols approved by the University of Science and Technology of China.

### 4.4 Preparation and Characterization of Engineered Probiotics

Probiotic Escherichia coli Nissle 1917 cells harvested at mid‐log phase were centrifuged, washed twice with PBS, and re‐suspended in 10 mM PBS (pH 8.5) to a density of 1 × 10^9^ CFU mL^−^^1^. Tannic acid was first added and the suspension was gently rotated at room temperature for 10 min in the dark to allow pre‐adsorption onto the bacterial surface. BPA and CaCl_2_ were then added sequentially, and incubation was continued for another 20 min. After reaction, free components were removed by centrifugation, and the resulting EP was washed three times with PBS and re‐suspended in sterile PBS for further use. The scanning electron microscope (SEM) images were taken on a JEOL JEM‐2200FS microscope. The BPA labeled with FITC and the expression of GFP was examined by using flow cytometry (Beckman CytoFlex, USA).

### 4.5 Growth Curves of Engineered Probiotics

Both untreated probiotics and BPA‐coated probiotics were diluted to reach an optical density (OD) value of ∼0.12 in LB, and incubated at 37°C with gently shaking. The OD value of cultures was recorded at 600 nm at 0.5 h intervals by microplate reader.

### 4.6 Flow‐Cytometry Analysis

After washing, FITC‐labeled BPA‐coated bacteria and simple‐mix controls were analyzed by flow cytometry using 488 nm excitation and FITC emission detection. Co‐culture engineered probiotic (EP) bacteria with tumor cells at an appropriate ratio for 3 h. Include untreated bacteria alone and free boronophenylalanine (BPA) at an equivalent boron dose as controls. Expose the co‐culture system to neutron irradiation. After irradiation, continue incubation for 12 h, stain with Annexin V‐FITC/PI, and analyze apoptosis by flow cytometry (excitation 488 nm, emission 530/615 nm).

### 4.7 Inductively Coupled Plasma‐Mass Spectrometry (ICP‐MS)

BPA‐coated and probiotics mixed with BPA groups were digested with concentrated nitric acid at 90°C until clear. The digested solutions were diluted with ultrapure water, and boron content was quantified using an ICP‐MS instrument. The boron loading capacity was calculated as the amount of boron. EP were incubated in 1 mL PBS at 37°C for 30 min with mild shaking (100 rpm). After centrifugation, the supernatant was collected and the cells were washed once with PBS. Boron contents in both supernatant and pellet were quantified by ICP‐MS. Release percentage was calculated as (B in supernatant)/ (total B) × 100%.

### 4.8 3D Tumor Spheroid Assay

B16‐luc cells were seeded at 2 × 10^3^ cells/well in 96‐well ultra‐low attachment plates and cultured to form tumor spheroids with a diameter of ≈200 µm. After co‐culturing Cy3‐labeled probiotics (group P and group EP) with the tumor spheroids for 1 h, neutron irradiation was performed. After an additional 5 h of co‐culture, imaging was performed using a confocal microscope to observe the infiltration depth of bacteria within the tumor spheroids. After washing the tumor spheroids three times with PBS, colony‐forming unit (CFU) counting was carried out to count the number of bacteria in individual tumor spheroids.

### 4.9 Tumor Model Establishment

To evaluate in vivo tumor growth, subcutaneous tumor models were established using B16 or 4T1 cells. C57BL/6 mice were inoculated subcutaneously with 1 × 10^6^ B16 cells per mouse, and BALB/c mice were inoculated subcutaneously with 1 × 10^6^ 4T1 cells per mouse. When tumor volumes reached approximately 150 mm^3^, tumor‐bearing mice were randomly assigned to different treatment groups, with six mice in each group.

### 4.10 In Vivo Bacterial Colonization Assay

For neutron irradiation, mice were positioned in the thermal neutron beam aperture of the facility. The thermal neutron flux was 5 × 10^7^ n/cm^2^/s, with a gamma‐ray dose rate of 4 Gy/h. Irradiation was performed for 3 h, delivering a total physical dose of 12 Gy to the tumor. For X‑ray irradiation, mice were exposed to 12 Gy of X‑rays at a dose rate of 2 Gy/min using an irradiator, with lead shielding to protect the body outside the tumor region. At the indicated time points post‑irradiation, tumors were harvested, homogenized, serially diluted, and plated on LB agar plates for colony‑forming unit (CFU) enumeration.

### 4.11 Co‐Cluture

Tumor cells were seeded in culture plates and divided into two groups: normoxic (21% O_2_) and hypoxic (1% O_2_) conditions. After 12 h of pre‐incubation under the respective oxygen conditions, cells were treated with either sorbitol‐dissolved BPA or bacteria‐modified BPA (designated EP) for an additional 3 h, with incubation continued under the corresponding normoxic or hypoxic atmosphere. Following drug treatment, culture supernatants were removed and cell monolayers were washed three times with ice‐cold PBS to remove residual extracellular drug. Cells were then harvested by trypsinization and collected by centrifugation at 180 g for 5 min. The cell pellets were resuspended in PBS and centrifuged again; this wash–centrifugation cycle was repeated five times to thoroughly remove unbound and free EP particles. After the final wash, cell numbers were quantified using a hemocytometer or automated cell counter. Cell pellets were then digested with concentrated nitric acid for complete sample dissolution. Intracellular boron concentrations were measured by inductively coupled plasma mass spectrometry (ICP‐MS), and results were normalized to cell number and expressed as boron mass per million cells.

### 4.12 BNCT Treatments

A D–D neutron‐generator accelerator‐based neutron source developed by the Institute of Energy, Hefei Comprehensive National Science Center, was employed for neutron‐irradiation therapy of tumor cells and mouse models. Fast neutrons of 180 kv produced via the D–D reaction were moderated by a machined polyethylene assembly to generate the thermal‐neutron beam required for the experiment. Throughout the treatment, mice were secured in a self‐developed, non‐anesthetic awake‐fixation device (Figure S4) that was precisely mounted at the irradiation port; the animals were positioned with their tails aligned to the beam axis so that only the tumor tissue was exposed to thermal neutrons while the remainder of the body remained outside the irradiated volume, ensuring accurate targeting. The tumor site and cells received a 4 h irradiation at a thermal‐neutron fluence rate of 5 × 10^7^ n cm^−^^2^ s^−^^1^. Borated polyethylene arranged around the moderator absorbed scattered neutrons, minimizing peripheral neutron background. During the 4‐h irradiation procedure, animal welfare was rigorously maintained. Mice were housed in a specialized restraint device that permitted limited lateral movement. The apparatus ensured excellent ventilation in the head region and provided a thermoregulated environment. To minimize stress and discomfort, the fixation was gently released for a 15‐min activity period at the end of each hour.

### 4.13 Tumor‐Volume Measurements

The vertical diameter of the tumor was measured using a vernier caliper, and the tumor volume was estimated by the formula V = 0.5 × A × B^2^ (where A represents the longer diameter and B represents the shorter diameter). The tumor volume was evaluated every other day. For statistical differences between the mean tumor growth curves, two‐way analysis of variance (two‐way ANOVA) combined with Bonferroni correction was used, with time and volume as variables.

### 4.14 Immune Microenvironment Analysis

Tumors were minced and digested with collagenase IV (1 mg mL^−^^1^) plus DNase I (100 µg mL^−^^1^) at 37°C for 1 h, the suspension was passed through a 70 µM strainer to obtain single cells. After red‐blood‐cell lysis, 1 × 10^6^ cells per tube were stained with fluorescently labeled antibodies, and dead cells were excluded using Fixable Viability Dye. Data was acquired on a CytoFLEX S cytometer and analyzed with FlowJo v10.8. Spleens were processed identically: organs were placed on a 70 µM screen, flushed with RPMI‐1640, gently ground to generate a single‐cell suspension, and subjected to the same staining and flow‐cytometry workflow.

### 4.15 In Vivo Imaging

To evaluate the in vivo distribution and targeting efficacy of engineered probiotics, a tumor‐bearing mouse model carrying B16 and 4T1 cells was adopted in the experiment. Specifically, B16 and 4T1 cells were first transfected with luciferase lentivirus respectively, and then inoculated into the left hip of C57BL/6 mice and BALB/c mice. Approximately one week later, 200 microliters of luciferin substrate were intraperitoneally injected, and bioluminescent imaging was performed using an IVIS imaging system to confirm the tumor volume. When the tumor volume reached 150–150 cubic millimeters, the mice were intravenously injected with normal saline, BPA, P, or EP, respectively. Subsequently, imaging was conducted by using an IVIS imaging system.

### 4.16 HPLC Method

The instrument used was an Agilent high‐performance liquid chromatograph (Germany). Chromatographic separation was performed on an Acquity UPLC HSS T3 column (100 mm × 2.1 mm, 1.8 µM) (Waters Corporation, MA, USA) heated to 30°C. The mobile phase consisted of methanol: formic acid: water (85:15:0.2, v/v/v), with isocratic elution at 100% for 7 min at a flow rate of 2 mL/min. The autosampler temperature was set to 4°C, and the injection volume was 5 µL.

### 4.17 Statistics Analysis

Data are presented as mean ± s.d. Comparisons between two groups were performed using unpaired t‐tests; multiple groups were analyzed by one‐way ANOVA followed by Tukey's post hoc test. *p* < 0.05 was considered statistically significant.

## Author Contributions

G.Z.L., X.C.M., Y.Y., and Y.L. conceived the study and designed all experiments. Y.L., Y.Z.Q., C.C., and J.H.W. performed the core experiments, analyzed the data, and drafted the initial manuscript. S.B.H. and B.B.L. contributed to experimental design optimization and conducted validation studies. M.S.Y. and J.H. W. assisted with experimental operations and data curation. B.B.L., X.C. provided technical support and critical research resources. G.Z.L., Z.G.L., X.C.M., Y.Y., and Y.L. supervised the overall project, acquired funding, and revised the manuscript critically for important intellectual content. All authors reviewed, edited, and approved the final manuscript.

## Conflicts of Interest

The authors declare no conflicts of interest.

## Supporting information




**Supporting File**: advs77542‐sup‐0001‐SuppMat.docx.

## Data Availability

The data that support the findings of this study are available from the corresponding author upon reasonable request.
