# Supplementary material for: Radiation‐Triggered Chemokine‐Secreting Probiotics for Boron Neutron Capture Therapy Based Radioimmunotherapy
Source: Adv Sci (Weinh). 2026 Sep 1;13(62):e77542. doi: 10.1002/advs.77542 (PMC13534782; doi:10.1002/advs.77542)
Supplement: Supplementary file 1 [file ADVS-13-e77542-s001.docx]

Supplementary Materials

**Figure S1. Boron content in EP-co-incubated tumor cells.** Data are presented as mean ± s.d. (n = 3 mice per group). The data were analyzed by one way ANOVA with Holm–Šídák multiple-comparisons test. ***^ns^***p >0.05, *p < 0.05, **p < 0.01, ***p < 0.001, and ****p < 0.0001.

**Figure S2. Boron-10 concentrations in major organs and tumor tissues at 1 h post-injection.** Data are presented as mean ± s.d. (n = 4 mice per group).

**Figure S3. Bacterial retention in tumor spheroids.** CFU counts per spheroid after PBS washing and neutron irradiation.

**Figure S4** Anesthesia-free mouse irradiation device.
